# Laser Ablation of NiFe_2_O_4_ and CoFe_2_O_4_ Nanoparticles

**DOI:** 10.3390/nano12111872

**Published:** 2022-05-30

**Authors:** Erik Sachse, Marianela Escobar-Castillo, Friedrich Waag, Bilal Gökce, Soma Salamon, Joachim Landers, Heiko Wende, Doru C. Lupascu

**Affiliations:** 1 Institute for Materials Science and Center for Nanointegration Duisburg-Essen (CENIDE), University of Duisburg-Essen, 45141 Essen, Germany; erik.sachse@web.de (E.S.); doru.lupascu@uni-due.de (D.C.L.); 2 Technical Chemistry I and Center for Nanointegration Duisburg-Essen (CENIDE), University of Duisburg-Essen, 45141 Essen, Germany; friedrich.waag@uni-due.de (F.W.); goekce@uni-wuppertal.de (B.G.); 3 Materials Science and Additive Manufacturing, University of Wuppertal, 42119 Wuppertal, Germany; 4 Faculty of Physics and Center for Nanointegration Duisburg-Essen (CENIDE), University of Duisburg-Essen, 47057 Duisburg, Germany; soma.salamon@uni-due.de (S.S.); joachim.landers@uni-due.de (J.L.); heiko.wende@uni-due.de (H.W.)

**Keywords:** laser ablation, NiFe_2_O_4_, CoFe_2_O_4_, nanoparticles, Mössbauer, HGMS

## Abstract

Pulsed laser ablation in liquids was utilized to prepare NiFe_2_O_4_ (NFO) and CoFe_2_O_4_ (CFO) nanoparticles from ceramic targets. The morphology, crystallinity, composition, and particle size distribution of the colloids were investigated. We were able to identify decomposition products formed during the laser ablation process in water. Attempts to fractionate the nanoparticles using the high-gradient magnetic separation method were performed. The nanoparticles with crystallite sizes in the range of 5–100 nm possess superparamagnetic behavior and approximately 20 Am^2^/kg magnetization at room temperature. Their ability to absorb light in the visible range makes them potential candidates for catalysis applications in chemical reactions and in biomedicine.

## 1. Introduction

The steadily growing demand for nanomaterials in many industrial sectors requires a thorough understanding of suitable manufacturing processes. Laser-assisted synthesis for the production of nanoparticles (NPs) offers access to a variety of high purity materials. As the properties of NPs are inherently associated with their particle size, it is essential to control their size distribution [1]. Ferrite spinels have semiconducting properties with band gap energies in the visible light range, and are therefore of particular interest for photocatalytic water splitting and other catalytic applications. Laser ablation of solids in liquids (LAL) is a versatile and fast process for producing NPs in suspension [2]. There are almost no limitations in the selection of solids and liquids. In the past, a variety of ferrites and spinel-type oxides have been prepared as NPs in suspension via LAL. Tsuji et al. [3] published a study on the synthesis of spinel-type Co_3_O_4_ NPs by UV laser irradiation of Co, CoO, and Co_3_O_4_ microparticles suspended in water. In a follow-up study, the same group [4] demonstrated the synthesis of NPs of the mixed oxide spinel LiMn_2_O_4_ via a similar approach using IR laser irradiation. Relvas et al. [5] and Du et al. [6] generated photoluminescent NPs in suspension using LAL of doped mixed oxide spinels. Gopal et al. [7] investigated LAL of NiFe_2_O_4_ (NFO) and obtained NPs consisting of different phases, although no further investigation of the decomposition products was performed. Recently, Ozçelik et al. [8] used LAL of NFO targets to generate suspended NPs of mixed oxide spinel, which they investigated for both the photodegradation of dyes and for blood compatibility. Bulai et al. [9] tried to prepare CFO NPs using the LAL technique; however, they reported that the XRD pattern of the obtained NPs did not show any diffraction peaks, or only decomposition products were observed. In other studies, researchers have observed the laser-induced decomposition processes of mixed oxide spinels and ferrites; for example, Chan et al. [10] found NPs of Ni-doped γ-Al_2_O_3_, Al-doped NiO, and β-Ni(OH)_2_, in addition to NiAl_2+X_O_4_ micro fragments in suspension produced by laser irradiation of NiAl_2_O_4_ microparticles, in water. In another study, Waag et al. [11] observed the sequential decomposition of CFO to CoO, Fe-based layered double hydroxide, and possibly Fe_3_O_4_ or amorphous species during the laser irradiation of a continuous liquid jet of an aqueous CFO nanopowder suspension. In a recent study, Siebeneicher et al. [12] laser-processed aqueous suspensions of submicron BiFeO_3_ particles in two different liquid jet setups with a spherical and an elliptical cross-section of the jet. The authors achieved a more efficient size reduction of BiFeO_3_ particles when using the elliptical jet thanks to better illumination with laser light, although they found a higher proportion of decomposition products as well. NFO and CFO NPs are catalytic materials that are used for the preparation of different organic compounds [13,14], for hydrogen production [15,16], and in the nanotheranostics field as imaging and therapeutic agents [17,18,19,20].

The aim of this work was to use the LAL technique with a continuous liquid flow for the preparation of NFO and CFO NPs and to analyze the obtained products in terms of their composition, particle size distribution, crystallinity, magnetic properties, and light absorption behavior. For this purpose, crystal structure analysis, magnetometry, Mössbauer spectroscopy, and UV/VIS absorption and reflection measurements were performed. Furthermore, experiments with a High-Gradient Magnetic Separator were performed in order to separate smaller particles from larger ones. The magnetic properties and visible light absorption behavior of the obtained NPs make these materials interesting for many different kinds of applications.

## 2. Materials and Methods

### 2.1. Sample Preparation

NFO was prepared by the solid state method by mixing NiO (Alfa Aesar, Kandel, Germany, 99%) and Fe_2_O_3_ (Alfa Aesar, Kandel, Germany 99.9%) in stoichiometric proportions. The mixed powder was calcined for 2 h at 800 °C and 6 h at 1200 °C in air. CFO was prepared by the sol-gel method using cobalt acetate (Co(CH_3_COO)_2_.4H_2_O, Sigma Aldrich, Darmstadt, Germany, reagent grade), iron nitrate (Fe(NO_3_)_3_.9H_2_OSigma Aldrich, Darmstadt, Germany, 99.9%), and citric acid as starting reagents in a proportion of 1:2:3, respectively. These reagents were mixed in ethanol in a beaker and heated at 80 °C by continuous stirring until the solvent was completely evaporated. Then, the mixture was heated in an oven at 120 °C for 48 h. The dried material was ground in a mortar and calcined for 2 h at 700 °C. The calcined powders were ground again to destroy agglomerates. NFO and CFO ceramic targets were obtained from calcined powders at a sintering temperature of 1200 °C for 6 h and air conditions. The manufactured pellets had a diameter of 1.2 cm and an average thickness of 1.3 mm. For further analysis and laser ablation, the surface of the sintered pellets was manually ground and polished with SiC paper (Struers, Willich, Germany, 500 and 2400).

### 2.2. Laser Ablation

**Figure 1:**
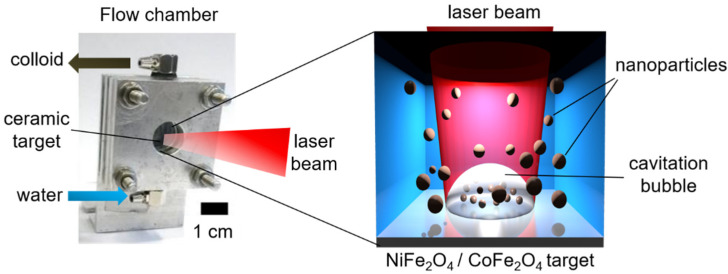
Experimental setup for the LAL preparation of NFO and CFO NPs under continuous liquid flow. The ceramic target is placed behind an optical window.

A single pellet was placed into a custom-built chamber behind an optical window (Figure 1). Water (MilliQ grade) continuously flowed through the chamber from bottom to top at a rate of 40 mL/min during the LAL of the pellet. A Nd:YAG solid-state laser (Atlantic, Ekspla, Vilnius, Lithuania) was used for LAL. The measured laser power was 14 W, the pulse repetition rate was 100 kHz, and the pulse duration was 10 ps. The laser beam was focused on the target surface by an F-theta lens with 100 mm focal length. The distance between the lens and the target surface was adjusted to have a maximized ablation rate. A galvanometer scanner was used to scan the target surface in a spiral pattern of 8 mm diameter at a speed of 4 m/s.

### 2.3. Analytic Methods

An XRD diffractometer (Panalytical Empyrean, Malvern, UK, Cu Kα radiation, step size of 0.05°) was used to analyze the crystal phase. Phase analysis was performed by Rietveld refinement using the High Score Plus program. The morphology and crystallinity of NPs were studied using SEM (Apreo S LoVac, Thermo Fischer Scientific, Dreieich, Germany) and high-resolution TEM (JEM-2200FS, JEOL, Freising, Germany). For the particle size distribution, we used ImageJ and Origin software for the particle counting and distribution plot, respectively. Magnetic field dependent M(H) curves (Quantum Design PPMS DynaCool, San Diego, CA, USA) of the pulverized pellets and the NPs were recorded via vibrating sample magnetometry at 300 K and 4 K up to a maximum magnetic field of 9 T. Mössbauer studies were recorded in transmission geometry at 300 K and 4 K using a helium bath cryostat, with 20 mg/cm^2^ of powder used for measurements. The radioactive source material was ^57^Co (Rh), mounted in a Mössbauer velocity transducer (WissEl GmbH, Ortenberg, Germany) operating in constant acceleration mode. Extinction and reflection measurements were performed using a UV-Vis spectrometer (Evolution 201, Thermo Fisher Scientific, Dreieich, Germany) and quartz glass cuvettes with a 10 mm beamway. For the HGMS experiments, an autoMACS column (Miltenyi Biotec, Bergisch Gladbach, Germany) was used. The separation column was placed between two neodymium disc magnets. The distance between the magnets was 1.25 cm, which roughly corresponds to the diameter of the column. The field strength between the magnets was determined to be about 0.2 T. For the experiments, 30 mL of the colloid were pumped into the column placed between the permanent magnets and the fluid was collected at the end of the column. Then, the column (without magnets) was flushed with the same volume of millipore water. For the long-term HGMS test, 2200 mL of colloid was pumped through the column.

## 3. Results

### 3.1. Optimization of Laser Ablation Parameters

Preliminary experiments were performed in order to ensure the optimal laser ablation conditions in terms of productivity. The highest removal rate was determined by varying the distance from the target surface to the focal position of the laser beam. UV-Vis extinction measurements were used to check the concentration of the obtained colloids, as these are directly proportional to the extinction values. The distance with the highest ablation rate (i.e., highest extinction value) was 0.5 mm away from the focal plane, having the target surface closer to the lens (Figure S1). The volume flow of the liquid medium was varied in order to further increase the productivity thanks to reduced shielding effects caused by particles and persistent bubbles. Through the variation of the flow rate in the ablation chamber (from 10 to 100 mL/ min) an optimum of 40 mL/ min was determined (Figure S2). The ablated masses of NFO and CFO after 52.5 min were almost the same (Table 1), indicating that the density of target material, which was determined by the Archimedes method, does not have a major influence on the ablation rate under these conditions.

### 3.2. Material Structure Analysis

**Figure 2:**
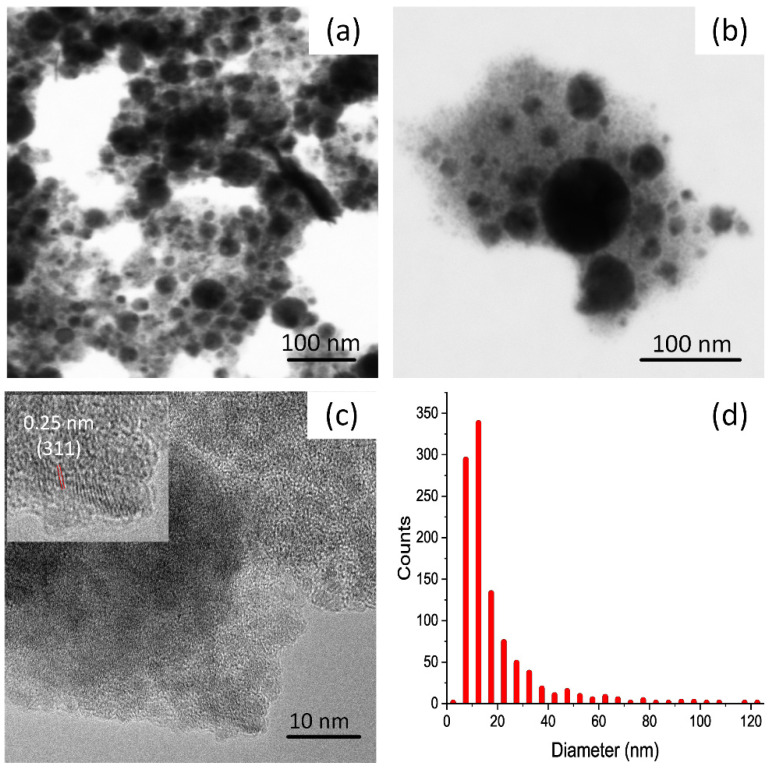
SEM image of NFO NPs (**a**), CFO NPs (**b**), HR-TEM image of NFO NPs (**c**) and particle size distribution of NFO NPs (**d**) obtained from the SEM image.

**Figure 3:**
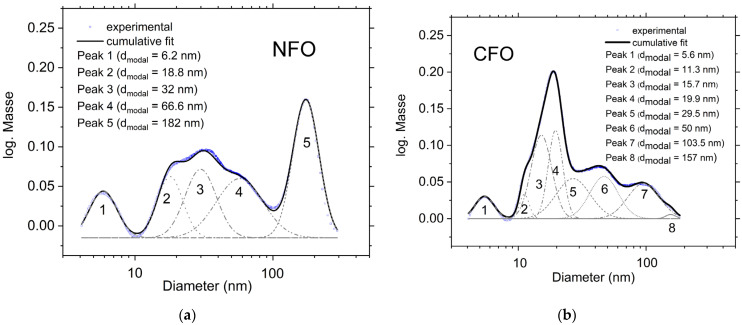
Mass-weighted particle size distribution of the NFO (**a**) and CFO (**b**) NPs, obtained using an analytical centrifuge.

Figure 2a shows an SEM image of NFO NPs. It can be seen from the particle size distribution (Figure 2d) that most of the particles are smaller than 20 nm in diameter. It is clear in the picture that there are many particles smaller than 10 nm, and possibly even smaller than 5 nm. These particles are strongly underrepresented in the particle size distribution, as the resolution for such particles is blurred. Furthermore, individual spheres with bigger sizes are present as well. The presence of such spherical species has been observed in different works for different ablated nanoparticles [2,12,21,22]. Its formation has been investigated by different authors [2,22], and is probably caused by different particle formation mechanisms during LAL, such as thermal vaporization and explosive boiling or the inhomogeneous distribution of beam intensity on the target material originating from different fluence regimes on the irradiated area, etc. The hydrodynamic diameter of the NPs was determined using an analytical centrifuge wiper (Figure 3a). The multimodal size distributions were fitted with a logarithmic normal distribution. In general, the obtained hydrodynamic diameter is bigger than the diameter obtained from microscopy images. Here, it can be seen that there is a significant fraction of particles with diameters smaller than 10 nm and that the majority of the particles are smaller than 100 nm. The relatively high peak (5) represents particles with a diameter bigger than 100 nm, however, this fraction can be caused by the detection of agglomerated particles.

EDX scans of selected areas confirm the expected molar proportions of Ni:Fe:O (1:2:4). The atom% values are 14.1 ± 0.1, 27.5 ± 0.3 and 58.3 ± 0.3 for Ni, Fe, and O, respectively. Elemental mapping images from a larger area (Figure S3) show that Ni, Fe, and O are homogeneously dispersed in these areas. An HR-TEM image of NFO NPs (Figure 2c) shows the crystalline structure of several individual particles, from which we were able to identify the crystal lattice spacing of 0.25 nm that corresponds to the (311) crystal plane of NFO [23].

In the SEM image of CFO NPs, the fraction of particles smaller than 10 nm is very high (Figure 2b), higher than in the case of the NFO sample. A particle size distribution of this material obtained from the analytical centrifuge is represented in Figure 3b. The presence of a particle fraction smaller than 10 nm was detected with this method, and it can be seen that the biggest particle fraction is in the range of 10–20 nm. The particle fraction larger than 100 nm is small, which is in accordance with the SEM results. The EDX element mapping scan of a selected area (Figure S4) shows the presence of Co, Fe, and O homogeneously dispersed within the powder.

**Figure 4:**
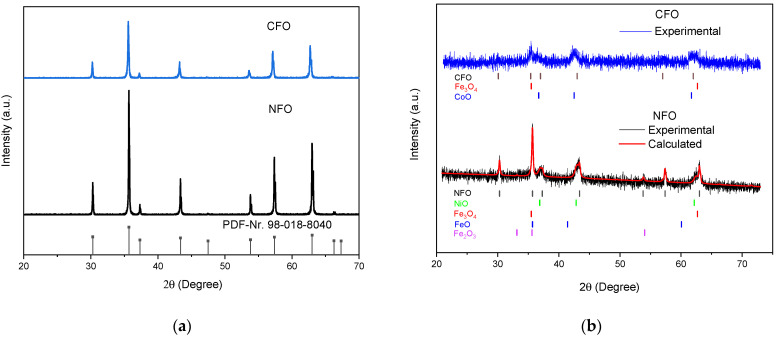
XRD diffractograms of NFO and CFO ceramic targets (**a**) and the obtained NPs (**b**). Perpendicular lines indicate the XRD peak positions of most intense peaks from PDF data: 98–018-8040 (NFO), 98–003-9131 (CFO), 98–007-7588 (Fe_3_O_4_), 98–007-1194 (Fe_2_O_3_), 98–067-1425 (NiO), 98–005-3519 (FeO), 98–002-8506 (CoO).

The crystal structure of the materials was analyzed using X-ray powder diffraction. The XRD diffractograms of the NFO and CFO ceramic targets in Figure 4a are in accordance with ICDD cards Nr. 98-018-8040 and 98-003-9131, respectively, which correspond to the cubic spinel structure. No secondary phases were detected in either target. In addition, the XRD diffractograms of the NPs (Figure 4b) show these characteristic reflexes. The noisy diffractograms with broadened peaks and low intensities are typical for nanoparticles with few nanometers size; especially for the CFO sample, the intensities of the peaks are very low, indicating a larger content of amorphous particles in this sample. This hinders the identification of formed byproducts, affecting the accuracy of the phase analysis. Although the EDX studies confirm the homogeneous distribution of elements in the NFO and CFO samples, the Rietveld analysis of XRD data indicates decomposition products in the nanopowders. The obtained NFO NPs consist of approximately 50% NFO, 42% NiO, 6% Fe_3_O_4_, 3% Fe_2_O_3_, and approximately 1% FeO. These results can have a large error due to the noisy spectra, low intensity of the peaks, and because the signals of NFO and Fe_3_O_4_ are very close together. In the case of CFO NPs, a reliable Rietveld analysis from the XRD spectrum was not possible, however, from the peaks at 43° and 63° (Figure 4b), it is clear that additional reflexes are present, which could correspond to CoO and Fe_3_O_4_. Other impurities such as wustite (FeO) and hematite (Fe_2_O_3_), with main peaks at around 41° and 33°, respectively, could not be identified due to the high noise levels and low intensity of the peaks. From XRD data, the crystallite size of the NPs was calculated using the Scherrer equation to be approximately 18 nm for the NFO sample and 16 nm for the CFO sample.

### 3.3. Mössbauer Spectroscopy

**Figure 5:**
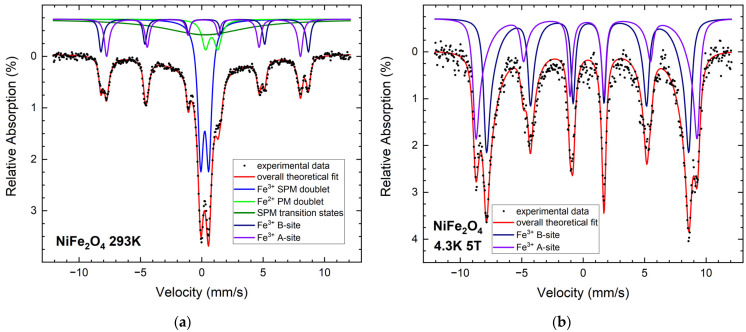
Mössbauer spectra of NFO NPs at 293 K (**a**) and at 4.3 K with a magnetic field of 5 T applied parallel to γ-ray propagation direction (**b**).

**Figure 6:**
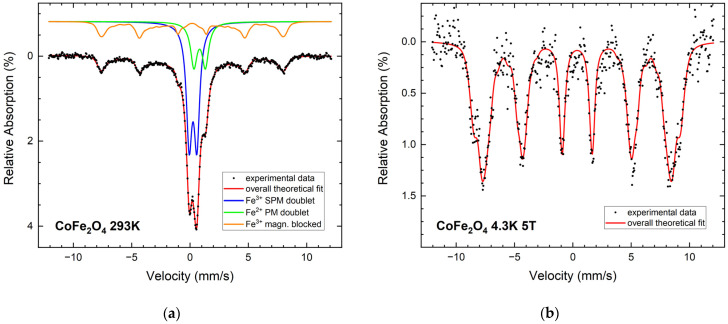
Mössbauer spectra of CFO NPs at 293 K (**a**) and 4.3 K (**b**) with 5 T magnetic field.

In order to investigate the magnetic properties of the NPs and evaluate the Fe oxidation state, Mössbauer spectroscopy was performed, as summarized in Figure 5 and Figure 6. For NFO NPs, the room temperature spectrum (Figure 5a) displays a dominant doublet sub-spectrum with approximately 39% spectral area, which is most probably caused by the smallest particles showing superparamagnetic behavior. In addition, the asymmetric structure in the center of the spectrum indicates the presence of an additional smaller doublet of higher isomer shift close to 0.95 mm/s, which points towards an Fe^2+^ compound, probably wustite (FeO), as discussed below. To verify this assumption, spectra at lower temperatures were recorded, as shown in Figure S5. It can be seen that this doublet contribution is present down to ca. 200 K, while it disappears at lower temperatures. It is reasonable to assume that this contribution is in a magnetically ordered state below 200 K, where the limited spectral area of only about 10% hinders the observation of the corresponding sextet subspectrum. As the Néel temperature of wustite NPs is around 200 K [24], this observation supports the assumption that this doublet belongs to Fe^2+^ in wustite. Beginning superspin fluctuation in intermediate sized particles results in broadened transition states (olive). Smaller magnetically blocked sextets comprising ca. 17% and 14% of the relative spectral area at room temperature, respectively, can be assigned to Fe^3+^ on the A- and B-lattice positions of the ferrimagnetic spinel AB_2_O_4_. It is difficult to resolve the contributions of, e.g., Fe_3_O_4_ and NiFe_2_O_4_ due to their generally similar hyperfine parameters and deformation of the spectrum by superparamagnetic relaxation. Studying a spectrum recorded at 4.3 K and 5 T (Figure 5b), the A- and B-sites can be resolved more clearly, although even here the lines display a slight asymmetry, presumably due to diminished hyperfine magnetic fields in Fe surface states and superposition with a minor Fe^2+^ contribution. To take this into account, A- and B-site subspectrum have been reproduced using distributions of hyperfine magnetic fields. A high degree of spin canting is seen in the NFO sample, visible by the minor reduction in the intensity of lines 2 and 5 after applying a magnetic field of 5 T [25].

Similar to NFO, the Mössbauer spectrum of the CFO sample (Figure 6a) shows a large doublet (50%) with parameters typical for superparamagnetic NPs. In addition, the paramagnetic wustite-like phase appears again, with 21%. Its share is significantly higher in comparison to the NFO sample, indicating a higher degree of decomposition of the CFO ceramic target during LAL. The weak intensity of the sextet is a clear indication of the small proportion of magnetically blocked CFO particles in the sample, hindering the resolution into A- and B-site contributions. Taking into consideration the comparatively high magnetocrystalline anisotropy of CFO, this points towards a high fraction of ultrasmall CFO nanoparticles. In-field analysis of the particles (Figure 6b) displays marginal alignment of magnetic moments along the field direction; A- and B-subspectra cannot be completely resolved, hindering the determination of Fe^3+^ ion occupation of the individual spinel lattice positions.

### 3.4. Magnetic Measurements

**Figure 7:**
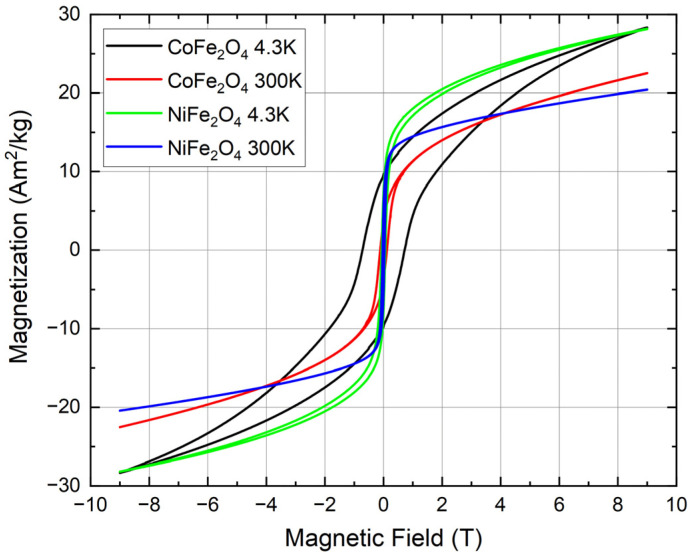
Magnetic hysteresis of NFO and CFO NPs at 4.3 K and 300 K.

Magnetization curves of the NFO and CFO NPs as a function of the external magnetic field are shown in Figure 7. The magnetic measurements of the ceramic targets can be seen in Figure S6 for comparison, with coercive fields and remnant magnetizations summarized in Table 2. No saturation of the magnetization curves was observed, with the magnetization increasing continuously upon rising fields up to 9 T, which can be explained due to spin frustration, as is clearly visible in the in-field Mössbauer experiments shown in Figure 5b and Figure 6b. An additional minor contribution could originate from the wustite-like byphase, as this is paramagnetic at room temperature. The maximum magnetization of the NPs is smaller compared to bulk reference samples, while the coercive field and the remnant magnetization in NFO NPs are higher than in their bulk counterparts. Relative to the ceramic bulk samples, a stronger decrease in magnetization can be observed in the CFO NPs compared to NFO, matching the higher spin frustration visible in the 5 T Mössbauer spectra (Figure 5b and Figure 6b).

### 3.5. Light Absorption Behavior

**Figure 8:**
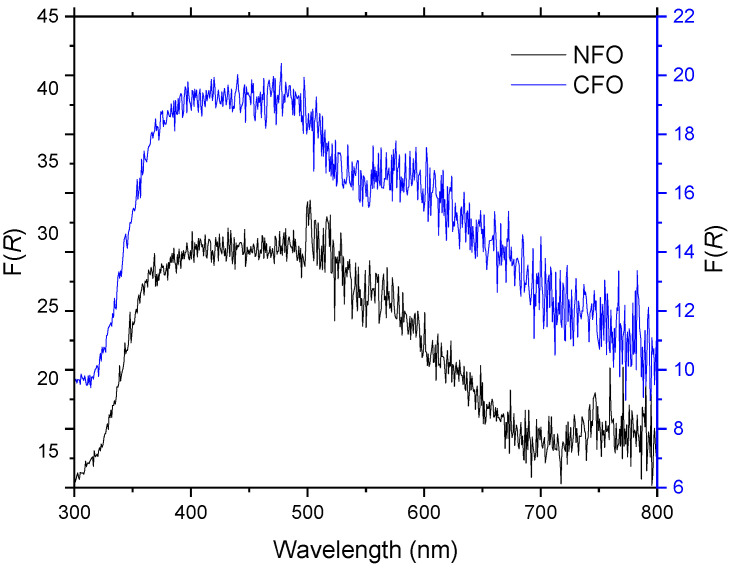
Kubelka–Munk function F(*R*) obtained from UV-Vis diffuse reflectance spectra of the NFO and CFO NPs.

In order to investigate the light absorption behavior of the NPs, diffuse reflection spectra were recorded with a UV-Vis spectrometer (Figure 8). The Kubelka–Munk function (Equation (1)) was used to calculate the function$\;F\left(R_{\infty }\right)$, which is proportional to the absorption coefficient α (where $R_{\infty }\;$represents the diffuse reflectance of a layer with infinite thickness). We observed that the NFO sample exhibits an absorption edge at around 700 nm, whereas the CFO sample shows a continuous absorption of light starting from 800 nm to around 400 nm. This confirms that both materials are optically active under visible light and underlines their potential application as catalysts.

(1) $$F\left(R_{\infty }\right)=\frac{{\left(1-R_{\infty }\right)}^{2}}{2R_{\infty }}$$

### 3.6. High-Gradient Magnetic Separation (HGMS)

The separation of magnetic particles from a solution can be performed using an external magnetic field. HGMS is a proven method whereby a magnetic field is typically applied across matrices of a ferromagnetic material to establish a high-field gradient. The separation process is strongly dependent on the particle size, its magnetic properties, and the strength of the applied magnetic field. Yavuz et al. [26] could successfully separate 6 nm from 12 nm particles from a Fe_3_O_4_ colloid using HGMS by varying the magnetic field strength, and Garcia et al. demonstrated the effective separation of superparamagnetic NPs using this technique [27].

**Figure 9:**
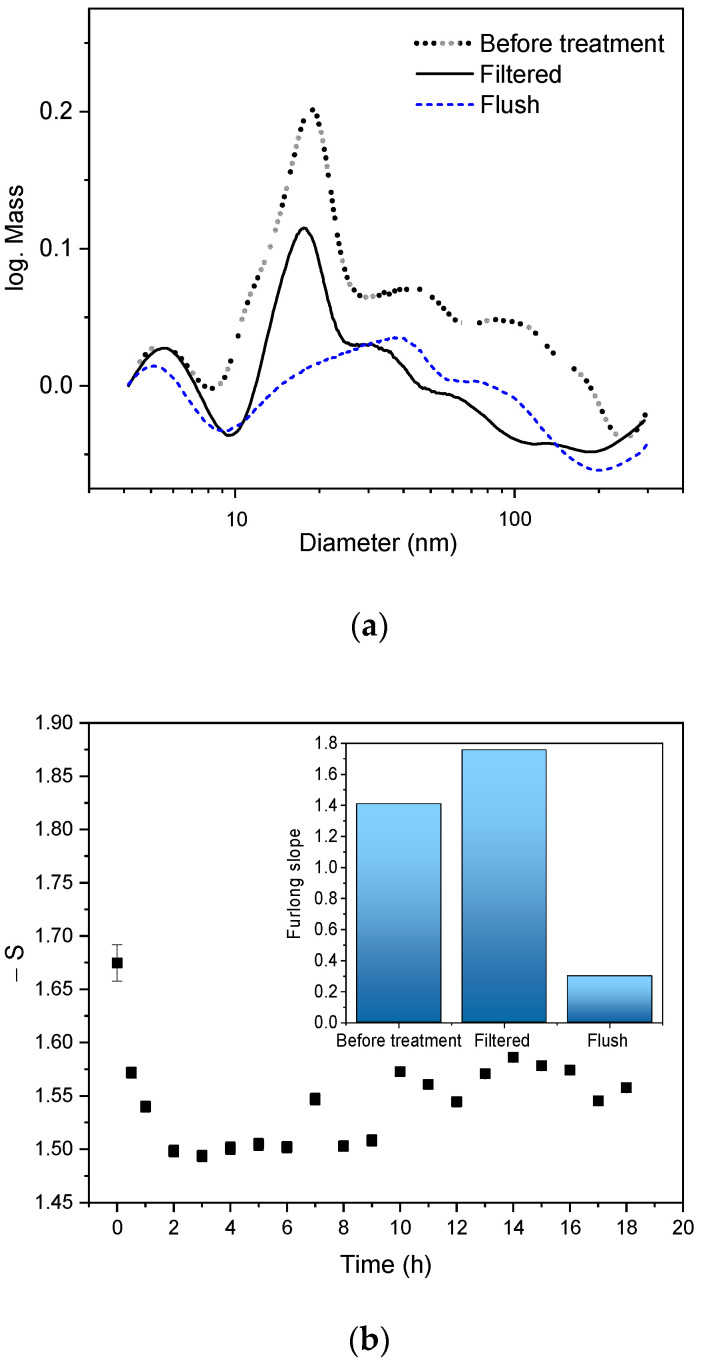
(**a**) Mass-weighted particle size distribution of the CFO NPs obtained before and after the HGMS separation process using 30 mL CFO colloid. Filtered fraction: collected at the end of the separator column that was placed between two disc magnets. Flush fraction: magnetically retained NPs on the column. (**b**) Long-term Furlong slopes analysis of the filtered fractions using 2200 mL CFO colloid. Inset in (**b**) shows the values of the fractions before treatment, from the filtered fraction (few mL fraction collected at 0 h), and from the flush fraction (retained NPs collected after 18 h).

We have tried to fractionate the small CFO NPs from the bigger ones using the HGMS method. The experiments were performed with a separator column and a 0.2 T magnetic field. The CFO colloid in water was introduced in the column with the constant flow (2 mL/min) under a magnetic field and collected at the end of the column. This collected colloid is called a “filtered” fraction. Then the magnets on the column were removed and the retained particles were collected with the same amount of solvent. This collected fraction is called “flush”. Figure 9a shows the obtained mass-weighted particle size distribution of the separated fractions using 30 mL CFO colloid. In the filtered fraction the number of particles bigger than 40 nm was reduced, indicating that with a magnetic field of 0.2 T, CFO NPs bigger than 40 nm were successfully retained in the column. In the flush fraction, it can be seen that the fraction of particles smaller than 30 nm is significantly reduced, while a particle fraction lower than 10 nm can be detected. This could be attributed to the strong agglomerated nature of the CFO sample, as shown in the SEM picture (Figure 2b). We assume that particles smaller than 10 nm are “stacked” on bigger particles, and were retained in the HGMS column as well.

For the long-term experiments, 2200 mL CFO colloid was pumped into the separation column. The Furlong slopes calculated from the UV-Vis extinction spectra of the filtered fractions can be seen in Figure 9b. The Furlong slope is an indicator for the size distribution [28] and was obtained by calculating the negative exponential slope of logarithmic extinction spectra (Equation (2)) and subsequent linear fit of the spectra in the range of log wavelengths of 2.35 and 2.5 log nm. A high (negative) Furlong slope value indicates a high amount of small particles in the colloid. The inset of Figure 9b shows the Furlong slope values of the untreated sample (1.41), the first filtered fraction (1.67, collected at 0 h), and the flush fraction after 18 h (0.3). A higher Furlong slope value is observed for the first filtered fraction (1.67) in comparison to the untreated colloid (1.41). These values confirm a significant retention of large particles in the column by applying HGMS. Nevertheless, it is interesting to look at the temporal Furlong slope development during the separation process (Figure 9b). In the first two hours, a continuous decrease of the Furlong slope values can be observed, indicating that fewer large particles remain in the column. The column seems to become saturated in this period. Then, there is a steady state for approximately 7 h. The Furlong slope during this time interval is around 1.5, which is similar to the untreated colloid value of 1.42 indicating that the separation column does not work efficiently any more. After that, the Furlong slope increases again (more small particles appear in the filtered fractions). The reason for this increased value could be that the retained particle layer in the column densifies over time and the column can again retain larger particles more efficiently, or that the smaller “stacked” particles on the bigger particles retained on the column are rinsed off by the solvent over time.

(2) $$S=-\frac{d\left(\log E\right)}{d\left(\log\;\lambda \right)}$$

## 4. Discussion

In this paper, we have investigated the preparation of NFO and CFO NPs using the LAL technique on ceramic targets in water. XRD and TEM analysis indicate that the obtained NPs are partially amorphous and that byproducts such as Fe_3_O_4_, NiO, and CoO were present in the colloidal samples. In addition, the presence of paramagnetic wustite (FeO) can be deduced from Mössbauer spectra, indicating that LAL of NFO and CFO ceramic targets using water as solvent leads to partial decomposition of the target material. The broad particle size distribution in the samples (less than 5 nm up to bigger than 100 nm) was confirmed by SEM, analytical centrifugation, and Mössbauer spectroscopy. In the CFO sample, the fraction of fine particles (≤5 nm) was greater in comparison to the NFO sample. These small particles are highly aggregated with larger particles, as can be seen in the SEM image. Detailed studies of the NPs magnetic behavior were performed via Mössbauer measurements and magnetometry. A considerable fraction of the obtained NPs was superparamagnetic (39% in the NFO and 50% in the CFO samples), possessing a maximum magnetization of 21 and 23 Am^2^/kg at 300 K for NFO and CFO, respectively.

Using the HGMS process with a 0.2 T magnetic field, the CFO colloid was separated into two fractions. Analysis of the fractions with an analytical centrifuge and UV-Vis extinction measurements indicated that in the separation column, particles larger than 40 nm were successfully retained, although the presence of particles with a size smaller than 10 nm continued to be detected in this fraction. This effect is probably caused by the highly aggregated nature of such small particles with larger ones. Further optimization of this process could be possible using surfactants, which can improve the dispersion of the NPs and avoid their aggregation. A long-term study of the HGMS separation process showed that this process works efficiently only within the first 1–2 h; afterwards, the column appears to be saturated. However, the HGMS separation column is a good alternative for roughly separating smaller particles from larger ones in the CFO sample.

The NFO and CFO magnetic NPs from the LAL process can be used as catalysts in different fields, especially thanks to their property to absorb light in the visible range, which was proven by UV-Vis diffuse reflectance measurements. Many authors have confirmed this catalytic behavior from NFO and CFO as well as from CoO_x_ [29], NiO [30,31], and even a combination of NFO/ NiO, which has been used as a catalyst in water splitting [32] and as a photocatalyst in the degradation of water pollutants [33]. In addition, the crystalline–amorphous nature of the NFO and CFO NPs could be interesting for catalytic applications, as demonstrated recently by Shen et al. [34], who demonstrated improved electrocatalytic hydrogen production in a crystalline–amorphous CoSe/ CoP heterojunction structure in comparison with a crystalline–crystalline structure of the same material combination.

## Figures and Tables

**Table 1:** Calculated ablation rates and concentrations of the colloids at a volume flow of 40 mL/min.

Material	Density (g/cm^3^)	Mass Ablation (g)	Concentration (mg/L)	Ablation Rate (mg/min)
NiFe_2_O_4_ (NFO)	5.19	0.0679	32.33	1.29
CoFe_2_O_4_ (CFO)	4.72	0.0591	28.14	1.13

**Table 2:** Magnetic properties of ceramic targets (bulk) and NPs obtained at 300 K and 4 K.

Sample	*Hc* (4 K) (mT)	*Hc* (300 K) (mT)	*Mr* (4 K) (Am^2^/kg)	*Mr* (300 K) (Am^2^/kg)	*Mmax* (4 K) (Am^2^/kg)	*Mmax* (300 K) (Am^2^/kg)
NFO bulk	2.23	0.07	1.0	0.03	52	48
NFO NPs	63.0	34.5	7.57	5.65	28	21
CFO bulk	174.3	17.13	46.84	6.63	90	81
CFO NPs	719.35	114.96	9.66	3.77	28	23

## Data Availability

Data presented in this article are available at request from the corresponding author.

## Supplementary Materials

The following are available online at https://www.mdpi.com/article/10.3390/nano12111872/s1, Figure S1: Variation of the target surface distance from the focal position of the laser beam for the determination of the optimal distance in LAL process, Figure S2: Variation of the colloid volume flow to determine the best nanoparticles productivity, Figure S3: Element mapping of NFO NPs, Figure S4: Element mapping of CFO NPs, Figure S5: Mössbauer spectra of NFO recorded at temperatures between 4.3 K and 293 K, Figure S6: Magnetic measurements of NFO- and CFO ceramic targets at 4.3 K and 300 K.
