# Supplementary material for: Laser Ablation of NiFe2O4 and CoFe2O4 Nanoparticles
Source: Nanomaterials (Basel). 2022 May 30;12(11):1872. doi: 10.3390/nano12111872 (PMC9181974; doi:10.3390/nano12111872)
Supplement: Supplementary file 1 [file nanomaterials-12-01872-s001.zip › nanomaterials-1723962-supplementary.pdf]

Supplementary Materials

Laser Ablation of $NiFe_2O_4$ and $CoFe_2O_4$ Nanoparticles

Erik Sachse [1], Marianela Escobar-Castillo [1,*], Friedrich Waag [2], Bilal Gökce [2,3], Soma Salamon [4], Joachim Landers [4], Heiko Wende [4] and Doru C. Lupascu [1]

[1] Institute for Materials Science and Center for Nanointegration Duisburg-Essen (CENIDE), University of Duisburg-Essen, 45141 Essen, Germany; erik.sachse@web.de (E.S.); doru.lupascu@uni-due.de (D.C.L.)
[2] Technical Chemistry I and Center for Nanointegration Duisburg-Essen (CENIDE), University of Duisburg-Essen, 45141 Essen, Germany; friedrich.waag@uni-due.de (F.W.); goekce@uni-wuppertal.de (B.G.)
[3] Materials Science and Additive Manufacturing, University of Wuppertal, 42119 Wuppertal, Germany
[4] Faculty of Physics and Center for Nanointegration Duisburg-Essen (CENIDE), University of Duisburg-Essen, 47057 Duisburg, Germany; soma.salamon@uni-due.de (S.S.); joachim.landers@uni-due.de (J.L.); heiko.wende@uni-due.de (H.W.)
* Correspondence: marianela.escobar@uni-due.de

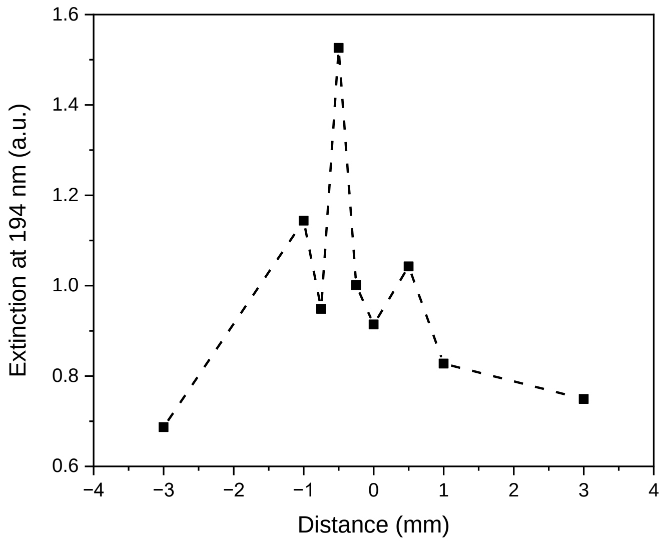

Figure S1. Variation of the target surface distance from the focal position of the laser beam for the determination of the optimal distance in LAL process.

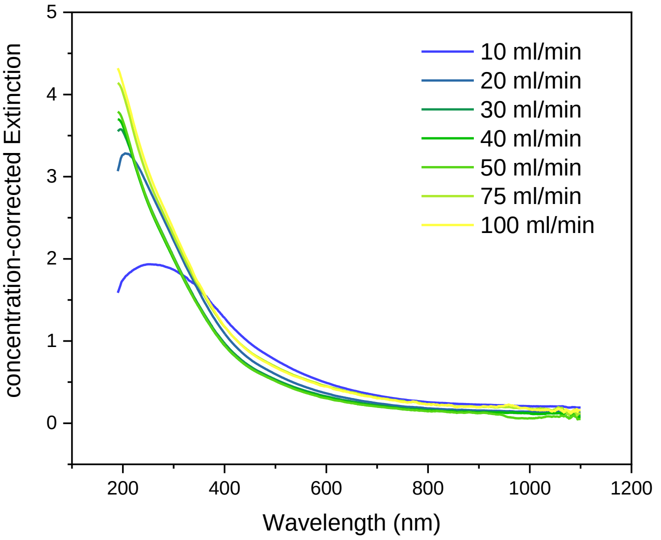

Figure S2. Variation of the colloid volume flow to determine the best nanoparticles productivity. Best volume flow at 40 ml/min, which shows high particle concentration with a curve maxima that is still in the measured range.

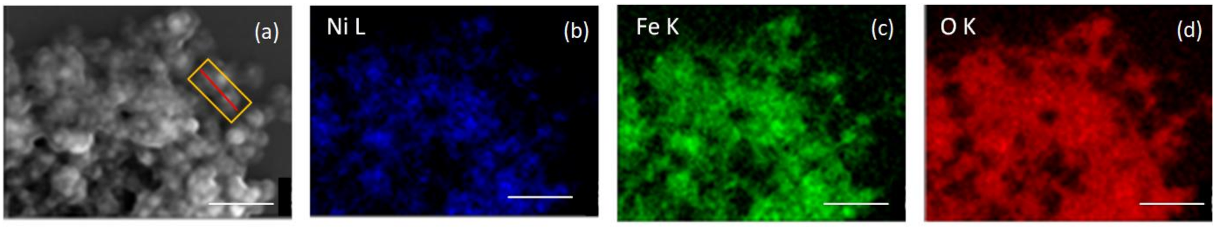

Figure S3. SEM Image of NFO NPs (**a**) and corresponding element mapping (**b,c,d**). Red marked line in (**a**) represent EDX scanned area to determine the Ni:Fe:O molar ratio. Obtained atom% values: 14.1 ± 0.1 for Ni, 27.5 ± 0.3 for Fe and 58.3 ± 0.3 for O. Bar represents 500nm.

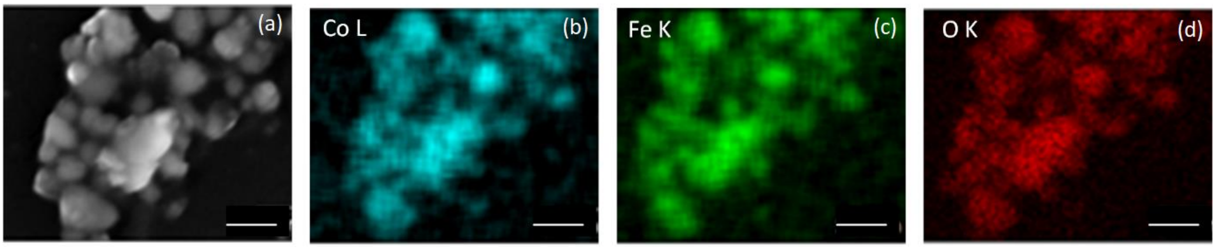

Figure S4. SEM image of CFO NPs (**a**) and corresponding element mapping (**b,c,d**). Bar represents 250nm.

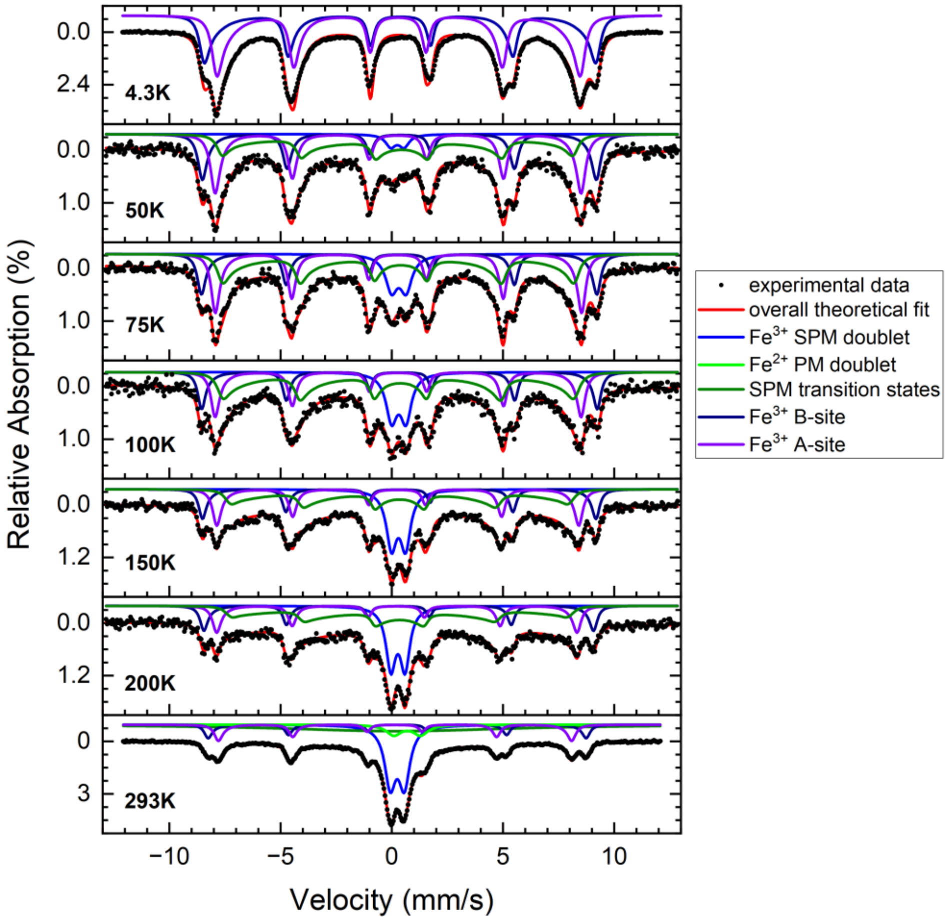

Figure S5. Mössbauer spectra of NFO recorded at temperatures between 4.3 K and 293 K in order to discern the temperature dependent evolution of Fe^{2+} contribution.

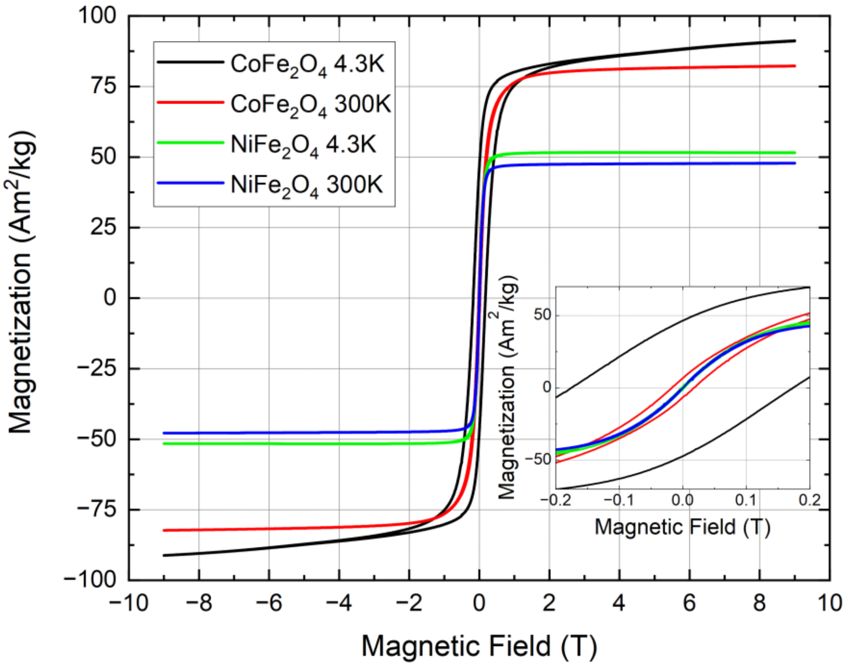

Figure S6. Magnetic measurements of NFO- and CFO ceramic targets at 4.3 K and 300 K.
